# Genome-Wide Association Study Reveals the Genetic Architecture Underlying Salt Tolerance-Related Traits in Rapeseed (*Brassica napus* L.)

**DOI:** 10.3389/fpls.2017.00593

**Published:** 2017-04-26

**Authors:** Heping Wan, Lunlin Chen, Jianbin Guo, Qun Li, Jing Wen, Bin Yi, Chaozhi Ma, Jinxing Tu, Tingdong Fu, Jinxiong Shen

**Affiliations:** National Key Laboratory of Crop Genetic Improvement/National Engineering Research Center of Rapeseed, College of Plant Science and Technology, Huazhong Agricultural UniversityWuhan, China

**Keywords:** rapeseed (*Brassica napus* L.), genome-wide association study (GWAS), salt tolerance, quantitative trait loci (QTL), single-nucleotide polymorphisms (SNP), candidate genes

## Abstract

Soil salinity is a serious threat to agriculture sustainability worldwide. Salt tolerance at the seedling stage is crucial for plant establishment and high yield in saline soils; however, little information is available on rapeseed (*Brassica napus* L.) salt tolerance. We evaluated salt tolerance in different rapeseed accessions and conducted a genome-wide association study (GWAS) to identify salt tolerance-related quantitative trait loci (QTL). A natural population comprising 368 *B*. *napus* cultivars and inbred lines was genotyped with a *Brassica* 60K Illumina Infinium SNP array. The results revealed that 75 single-nucleotide polymorphisms (SNPs) distributed across 14 chromosomes were associated with four salt tolerance-related traits. These SNPs integrated into 25 QTLs that explained 4.21–9.23% of the phenotypic variation in the cultivars. Additionally, 38 possible candidate genes were identified in genomic regions associated with salt tolerance indices. These genes fell into several functional groups that are associated with plant salt tolerance, including transcription factors, aquaporins, transporters, and enzymes. Thus, salt tolerance in rapeseed involves complex molecular mechanisms. Our results provide valuable information for studying the genetic control of salt tolerance in *B. napus* seedlings and may facilitate marker-based breeding for rapeseed salt tolerance.

## Introduction

Soil salinity is a major abiotic stress that threatens agricultural production all over the world (Zhu, 2001). Approximately 280 million hectares of agricultural land are affected by salt stress (Ruan et al., 2010). Due to traditional and unscientific irrigation management, soil salinity will become progressively more severe over time, and salt stress is predicted to affect over 50% of all agricultural land by 2050 (Flowers, 2004; Demiral and Türkan, 2006).

Plant response to salt stress is a complex genetic and physiological trait controlled by several quantitative trait loci (QTL) (Flowers, 2004). The response can be subdivided into two stages: early-occurring osmotic stress and accumulation of toxic ions (e.g., Na^+^ and Cl^−^ if in excess) (Munns and Tester, 2008). In the first stage, salt stress reduces soil water potential, thus decreasing root water-uptake ability. This water deficit leads to over-production of reactive oxygen species (ROS), including super oxides, hydrogen peroxide, and hydroxyl radicals (Mittler, 2002). Elevated ROS content disturbs normal cellular activity through oxidative damage of membrane lipids and proteins (Imlay, 2003). During the second stage, roots absorb Na^+^ at high concentrations; these ions are then transported to shoots later. Excess Na^+^ negatively affect plant growth rate and development, compromising metabolic processes and decreasing photosynthetic efficiency (Munns, 2005; Munns and Tester, 2008). Specifically, excess Na^+^ impedes K^+^ absorption, thus disrupting intracellular Na^+^ and K^+^ homeostasis, which is critical to plant growth and development (Schachtman and Liu, 1999; Shabala and Cuin, 2008; Ahmadi et al., 2011).

Saline land has traditionally been reclaimed through engineering technologies that repair salt-affected soil (Epstein et al., 1980). With recent biotechnological developments, plant scientists have increasingly favored breeding and screening salt-tolerant crop varieties through genetic modification as a cost-saving, convenient, and efficient alternative. Mapping the QTL of salt-related traits and cloning salt-tolerant genes have become effective approaches for coping with soil salinization in agricultural land. Conventionally, QTL mapping uses bi-parental populations to dissect the genetic mechanisms of salt stress-related traits (Kumar et al., 2015), resulting in the discovery of salt tolerance-linked QTLs from several crops. In rice, for example, Lin et al. (2004) mapped QTL for root and shoot Na^+^/K^+^ concentration and transportation to five chromosomes, while also cloning *SKC1*, a key gene that controls shoot K^+^ content. In soybean, a major QTL controlling salt tolerance was identified, accounting for 41, 60, and 79% of the total genetic variation for salt tolerance in field, greenhouse, and combined environments, respectively (Lee et al., 2004). Finally, in maize, Cui et al. (2015) detected 20 additive and nine epistatic QTLs for salt tolerance, based on SNP markers expressed at the seedling stage. With the recent introduction of genome-wide association studies (GWAS; also called association mapping) to crop genetic research, the utility of QTL has been further enhanced (Mackay and Powell, 2007; Cockram et al., 2010). Compared with traditional screening methods, genotyping, and sequencing technologies are more efficient (Zhu et al., 2008), making association mapping a widely used technique for dissecting salt-related traits in crops such as rice (Kumar et al., 2015), barley (Long et al., 2013), and soybean (Kan et al., 2015).

Rapeseed (*Brassica napus* L., genome AACC, 2n = 38) is one of the most important oil crops in the world. Annually, rapeseed oil production reaches 26 million tons, accounting for approximately 15% of global vegetable oil production (USDA ERS, 2014). Therefore, understanding the genetic architecture for a complex trait such as salt tolerance is crucial in the development of new, robust rapeseed cultivars. Recently, with the development of the *Brassica* 60K Infinium SNP array, association mapping has been successful in detecting QTL for complex traits in rapeseed. Recently, Li et al. (2014, 2016) identified 23 QTLs for erucic acid content, glucosinolate content, oil content, seed weight, plant height, and primary branch. Additionally, Xu et al. (2016) identified 35 QTLs for flowering time found near *Bn-scaff_16362_1-p380982*, approximately 13 kb from *BnaC09g41990D*, which is an orthologous to *A. thaliana CONSTANS* (CO), an important gene in the photoperiod flowering pathway. Liu et al. (2016) identified 50 QTLs for seed oil content that could explain approximately 80% of the total phenotypic variance in rapeseed, while Luo et al. (2015) identified nine SNPs for harvest index and seed yield per plant that explained 3.42% of the phenotypic variance in the former trait. Moreover, Sun et al. (2016a,b) identified 68 loci associated with plant height and 53 loci associated with branch angle in rapeseed. Yet despite the recent increase in rapeseed association mapping, limited QTLs for rapeseed salt tolerance have been reported.

In the present study, we examined a panel of 368 rapeseed accessions using 19,167 genomic SNPs from the Illumina *Brassica*SNP60 Bead Chip. Seedling growth-related traits from the panel were investigated in three environments. The objectives of this study were as follows: (i) obtain a better understanding the effect of salt stress on rapeseed seedlings, (ii) examine the relationships between salt stress-related traits in seedlings, and (iii) perform GWAS to identify salt-tolerance-related SNPs and possible candidate genes underlying salt tolerance at the seedling stage.

## Materials and methods

### Plant materials

The association panel used in this study consisted of 368 diverse rapeseed accessions derived from recently published studies (Liu et al., 2016; Xu et al., 2016). These germplasms originated from 10 countries on four continents (Table S1); most came from China and included winter oilseed rape (OSR) [26], semi-winter OSR [307], and spring OSR [35].

### Experimental design and trait measurement

To minimize environmental impact, rapeseed salt-tolerance traits were evaluated at the seedling stage using a previously described hydroponic system (Tocquin et al., 2003), with slight modifications to accommodate larger hydroponic containers (60× 40× 10 cm). Thirty healthy seeds from all 368 rapeseed lines were germinated for 7 d in a salver (60× 40× 10 cm) containing double-distilled water, separated into 96 plots with nylon rope, and covered with one sheet of medical gauze on the bottom. At the beginning of the 8th day, 10 similar seedlings per plot were selected and individually transferred to the hydroponic system for another 4 weeks of growth. According to the use of the nutrient solution method for rapeseed seedling growth in our laboratory, different concentrations of Hoagland solution (10 L) were used as the hydroponic growing medium: 0.25× for the first week, 0.5× for the second week, and 1× for the third to fourth weeks. We designed eight concentration gradients (0, 110, 140, 170, 200, 230, 260, 290 mM) NaCl to carry out salt stress treatment on rapeseed seedlings, and the results shown that the 230 mM NaCl was suitable to evaluated salt tolerance of rapeseed seedlings (Unpublished). Thus, at the beginning of the fifth week, 134 g NaCl was mixed with 10 L 1× Hoagland solution to generate a 230 mM NaCl solution, while 1× Hoagland solution without NaCl was used for the control condition and the NaCl and without NaCl solution were used to plant rapeseed seedling for another 2 weeks. Experimental and control 1× Hoagland solutions were replaced every 7 d.

The seedlings were harvested for measuring shoot length (SL), taproot length (RL), and shoot fresh weight (SFW). Harvested material from salt-stressed seedlings were washed with distilled water and oven-dried at 105°C for 30 min, then dried further at 80°C for 48 h. To determine shoot Na^+^ content in each rapeseed line, dried shoots from five replicates per line were pooled, placed in a 50-mL centrifuge tube containing two 6-mm steel balls, and pulverized for 5 min with a high-speed oscillator (model SK450, F&FM, Australia). Pulverized samples were then sieved (100 mesh). A 100 mg sample from each rapeseed line was placed in test tubes with 2 mL nitric acid, incubated in a 100°C water bath for 1 h, and then distilled water was added until a final volume of 10 mL was attained. A flame photometer (model FP640) was used for Na^+^ estimation (μg/mg dry weight), calculated with the following equation (Ammar et al., 2009):

$$\begin{array}{l} {\text{Na}}^{+}\text{concentration}\;\left(\mu \text{g}/\text{mg dwt}\right)\;=\;\left[\text{C}\;\left(\text{d}{\;}^{*}\;\text{V}/1000\right)\right]/\text{dwt} \end{array}$$

where C is the flame photometer reading, d is the dilution factor, V is extraction volume (mL), and dwt is the dry weight (mg).

The salt tolerance index was calculated following previous publications, as the ratio of experimental (saline) values to control values (Munns and James, 2003; Long et al., 2013). The hydroponics system used for testing consisted of 10 units and involved 10 plants grown separately in containers (five per condition), with 77 possible plant positions. Each plant represented one experimental unit. Thus, there were 10 replicates per genotype: five for the salt treatment and five for the control. Each experiment comprised 10 randomized units allocated to control (0 mM NaCl) and salt (230 mM NaCl) hydroponic treatment groups. The experiment was repeated three times, once in a natural outdoor environment during October 2014 and twice in a greenhouse (20°C ± 2) during November 2013 and September 2014.

### Phenotypic data analysis

Statistical analysis of all phenotypic data was performed in SPSS version 18.0 (IBM Corp, Armonk, NY, USA). Descriptive statistics and Pearson's correlations between traits were performed using the mean values of all phenotypic data from 368 rapeseed accessions. The frequency distribution of each trait was calculated in R (R Core Team, Vienna, Austria). The broad-sense heritability for the panel was calculated with the R package lme4 (Merk et al., 2012), using the equation *h*^2^ = o^,2^_*g*_/(o^,2^_*g*_ + o^,2^_*ge*_/*n* + o^,2^_*e*_/*nr*), where o^,2^_*g*_ is the genetic variance, o^,2^_*ge*_ is the genotype × environment interaction variance, o^,2^_*e*_ is the error variance, *n* is the number of environments, and *r* is the number of replications.

### Population structure and relative kinship analysis

The genotypes of the 368 rapeseed accessions in this study have been previously published (Liu et al., 2016; Xu et al., 2016) and were evaluated here using the *Brassica* 60 K Illumina® Infinium SNP array. Following previously described methods (Sun et al., 2016b), 19,167 high-quality SNPs (call rate > 0.7; SNP call frequency > 0.75; minor frequency > 0.05; AA, BB frequency > 0.03; and GenTrain score > 0.5) was used for population structure analysis and relative kinship analysis. The population structure was estimated in STRUCTURE 2.3.4 (Falush et al., 2003) with the Bayesian Markov Chain Monte Carlo (MCMC) model. The *K*-value representing the number of subgroups was set from 1 to 10, and five independent runs for each K were performed, with burn-in length and iterations both set to 100,000 under the admixture and correlated allele frequencies models. The optimal *K*-value was determined using the log likelihood of the date [LnP(D)] and ΔK based on the rate of change in LnP(D) between successive *K*-values, as previously described (Evanno et al., 2005). The Q matrix was obtained in CLUMPP (Jakobsson and Rosenberg, 2007), through integrating replicate runs from STRUCTURE. The relative kinship matrix (K matrix) was obtained using SPAGeDi (Hardy and Vekemans, 2002), and negative values between two rapeseed accessions were set to 0 (Yu et al., 2006).

### Genome-wide association analysis

Association mapping was conducted in TASSEL 4.0 (Bradbury et al., 2007) with four models: GLM without considering Q and K, GLM considering Q (Q), MLM considering K (K), and MLM considering Q and K (Q + K). The SNP markers that were significantly associated with the three salt tolerance indices (ST-RL, ST-SL, ST-SFW) and shoot Na^+^ content were identified according to a probability level of *P* ≤ 0.0001 or −logP ≥ 4.00, following existing published thresholds (Yong et al., 2015).

## Results

### Phenotypic variation among accessions for growth-related traits and salt tolerance index traits

**Figure 1:**
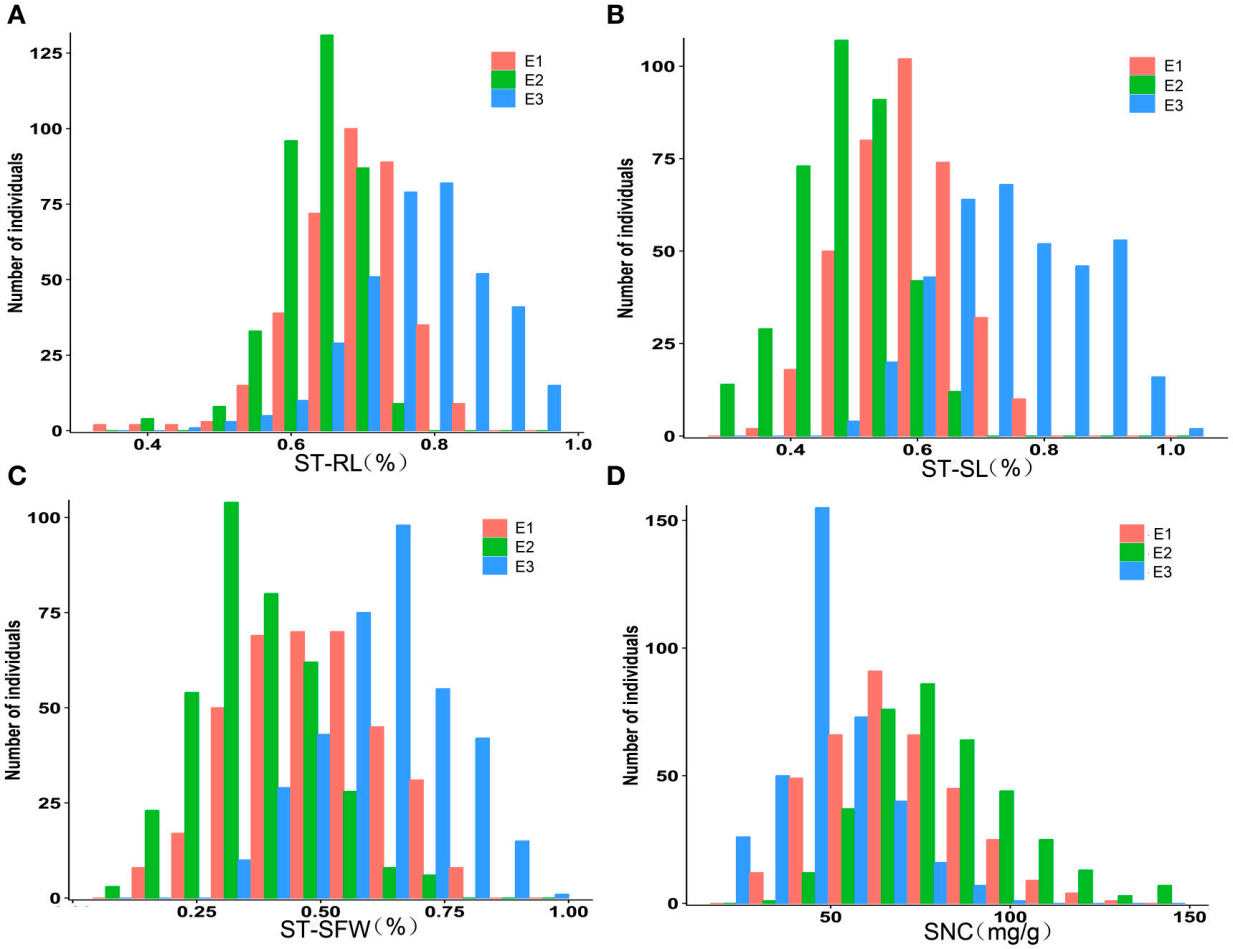
**Distribution of the three salt indices and SNC in the association panel of 368 accessions grown in three environments. (A)** ST-RL. **(B)** ST-SL. **(C)** ST-SFW. **(D)** SNC. E1, greenhouse in 2013; E2, greenhouse in 2014; E3, natural environment condition in 2014. ST, salt tolerance index; RL, root length; SL, shoot length; SFW, shoot fresh weight.

The 368 rapeseed accessions were planted in three environments (E1: greenhouse 2013, E2: greenhouse 2014, E3: natural environment 2014) and extensive phenotypic variation was observed for RL, SL, and SFW in salt stress and control conditions (Table 1, Figure S1, Data S1). Furthermore, salt stress significantly reduced RL, SL, and SFW compared with the control. To control for background differences, salt tolerance indices (ST) of RL, SL, and SFW were used in our evaluation of rapeseed salt stress response. Across the three environments, ST-RL, ST-SL, and ST-SFW varied between 0.370 and 0.960 (averages: 0.636 ± 0.058–0.776 ± 0.091), 0.310 and 0.930 (0.532 ± 0.080–0.715 ± 0.094), and 0.087 and 0.940 (0.374 ± 0.121–0.617 ± 0.132) (Figures 1A–C). Control growth traits had higher mean values and less variation than those measured under salt stress. In addition, salt-stressed shoot Na^+^ content (SNC) ranged 20.2–143.5 mg g^−1^ with averages of 47.7 ± 13.7–80.3 ± 21.3 mg g^−1^ in the three growth environments (Figure 1D). For the salt tolerance indices, the STs in the greenhouse were lower than that in outdoor, suggesting that the environment would affect the salt tolerance of rapeseed at seedling stage (Table 1). Heritability (*h*^2^) estimates of all traits ranged from 79.60% (control SFW) to 93.46% (ST-SL), suggesting their stable inheritance (Table 1).

**Table 1:** **Phenotypic variation in growth−related traits and salt tolerance index traits**.

**Trait**	**Treat**	**Env**	**Minimum**	**Maximum**	**Mean**	***SD***	**CV (%)**	**Skewness**	**Kurtosis**	***h*^2^ (%)**
RL	C	E1	19.2	36	27.1	2.28	8.41	0.43	1.22	82.27
		E2	20	35	25.8	1.96	7.60	0.46	1.00	
		E3	16.6	33	23.6	2.57	10.87	0.53	0.32	
	S	E1	10.2	26.5	18.7	2.30	12.31	−0.41	1.28	80.78
		E2	9.7	21.1	16.4	1.76	10.73	−0.26	0.70	
		E3	9.6	26	18.3	2.78	15.15	0.12	0.18	
	ST	E1	0.37	0.85	0.69	0.08	11.18	−0.95	2.07	87.37
		E2	0.38	0.76	0.64	0.06	9.06	−1.00	2.41	
		E3	0.47	0.96	0.78	0.09	11.79	−0.43	0.19	
SL	C	E1	19.1	39.2	27.9	3.46	12.40	0.18	−0.52	81.40
		E2	18.18	38.9	26.4	4.02	15.24	0.98	0.63	
		E3	16.4	34.1	24.9	3.78	15.19	0.02	−1.01	
	S	E1	9.9	24.2	16.3	2.16	13.29	0.08	0.52	86.40
		E2	7.2	20	12.7	2.13	16.76	0.09	0.30	
		E3	10.9	28.1	18.4	3.44	18.66	0.29	−0.33	
	ST	E1	0.37	0.8	0.59	0.09	14.52	−0.04	−0.31	93.46
		E2	0.27	0.65	0.49	0.08	16.33	−0.15	−0.27	
		E3	0.47	1	0.75	0.12	15.74	0.05	−0.90	
SFW	C	E1	5.05	18.59	9.66	2.61	27.01	0.67	0.00	79.60
		E2	4.46	19.62	10.71	2.67	24.92	0.26	−0.02	
		E3	2.85	12.32	7.26	1.86	25.60	0.16	−0.23	
	S	E1	0.85	11.04	4.63	1.68	36.36	0.60	0.79	90.09
		E2	0.79	9.43	3.97	1.52	38.28	0.51	0.40	
		E3	1.35	9.92	4.45	1.41	31.80	0.46	0.34	
	ST	E1	0.14	0.83	0.49	0.14	29.70	0.00	−0.50	92.31
		E2	0.09	0.75	0.37	0.12	32.41	0.36	−0.01	
		E3	0.29	0.95	0.62	0.13	21.35	−0.15	−0.38	
SNC		E1	29.6	133	68.72	18.71	27.23	0.45	0.07	91.02
		E2	35.6	145.3	81.28	20.26	24.93	0.60	0.36	
		E3	20	94.1	47.69	13.68	28.68	0.55	0.44	

*Trait: RL, root length; SL, shoot length; SFW, shoot fresh weight; ST, salt tolerance index; SNC, shoot Na^+^ content*.

*Treatment: C, no salt treatment; S, salt treatment; ST, salt tolerance index*.

*Environment: E1, greenhouse in 2013; E2, greenhouse in 2014; E3, natural environment in 2014*.

*SD, standard deviation; CV (%), coefficient of variation; h^2^, heritability of means*.

### Salt tolerance index and its correlation with other traits in rapeseed

Pearson's correlations between the three growth-related traits (under control or salt stress conditions) and various STs were calculated from 2013 greenhouse (Table 2). In the control, RL was not significantly correlated with SL or SFW; however, SL and SFW were positively correlated (*P* < 0.01). Under salt stress, SL was positively correlated with RL (*P* < 0.05) and SFW (*P* < 0.01). Further, ST-RL was negatively correlated with RL in control conditions (*P* < 0.01) and positively correlated with RL in salt stress conditions (*P* < 0.01). The same patterns were observed for ST-SFW and ST-SL, but not for ST-SL and SL, which were uncorrelated in the control. Furthermore, SNC under salt stress was not significantly correlated with any traits, indicating that rapeseed salt tolerance is not linked to Na^+^ content in shoots. Results were consistent across all environments (Table S2). As shown in Table S3, the correlations between phenotypic data in different environments were generally high, and majority reached to 0.8, indicating our screenings were reliable.

**Table 2:** **Phenotypic correlations between all of the salt tolerance traits based on the trait means of plants grown in the greenhouse in 2013**.

**Trait**	**CRL**	**TRL**	**ST-RL**	**CSL**	**TSL**	**ST-SL**	**CSFW**	**TSFW**	**ST-SFW**	**SNC**
CRL	1	0.451^**^	−0.249^**^	0.082	0.065	−0.001	0.086	0.089	0.029	0.066
TRL		1	0.748^**^	0.1	0.094	0.01	0.033	0.055	0.042	0.091
ST-RL			1	0.051	0.056	0.011	−0.027	−0.004	0.026	0.047
CSL				1	0.346^**^	−0.529^**^	0.654^**^	0.357^**^	−0.131^*^	0.037
TSL					1	0.605^**^	0.335^**^	0.605^**^	0.437^**^	−0.037
ST-SL						1	−0.247^**^	0.239^**^	0.504^**^	−0.063
CSFW							1	0.561^**^	−0.186^**^	0.01
TSFW								1	0.681^**^	−0.062
ST-SFW									1	−0.086
SNC										1

*Trait: CRL, root length under control treatment; CSL, shoot length under control treatment; CSFW, shoot fresh weight under control treatment; TRL, root length under salt treatment; TSL, shoot length under salt treatment; TSFW, shoot fresh weight under salt treatment; ST-RL, ST-SL, and ST-SFW, salt tolerance index of RL, SL, and SFW, respectively*.

* *P < 0.05*,

** *P < 0.01*.

### Population structure and genetic relatedness in rapeseed cultivars

**Figure 2:**
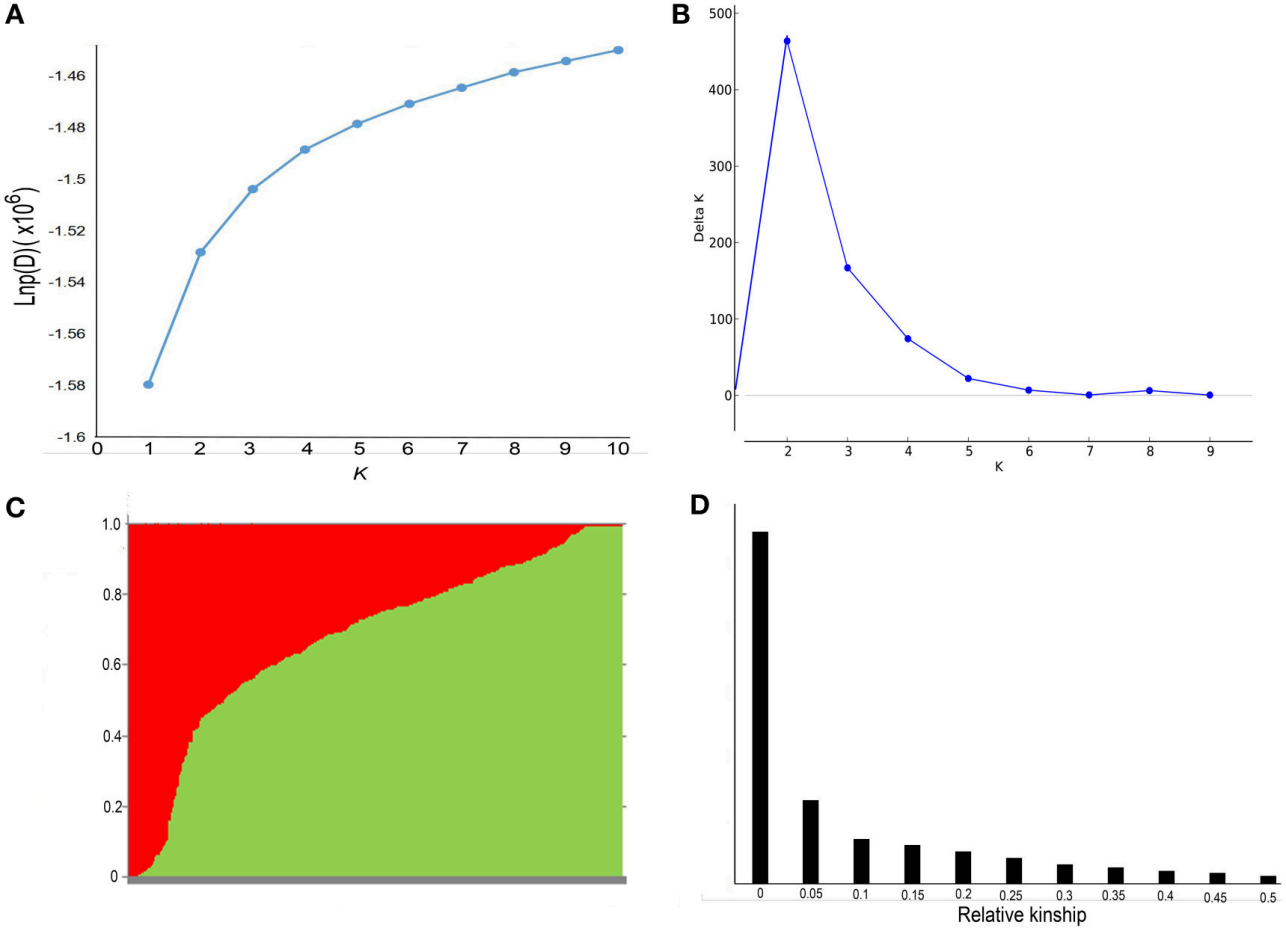
**Analysis of the population structure and relative kinships of the 368 rapeseed accessions. (A)** Estimated LnP(D) of possible clusters (K) from 1 to 10. **(B)** Δ*K* was based on the rate of change of LnP(D) between successive *K*-values. **(C)** Population structure based on *K* = 2. Red represents Subgroup Q1; green, Subgroup Q2. **(D)** Distribution of pairwise relative kinship estimated in the entire population P (368 rapeseed accessions). Only kinship values ranging from 0 to 0.5 are shown.

Population structure and genetic relatedness was calculated from 19,167 SNPs in the rapeseed accessions. As K increased from 1 to 10, LnP(D) increased continuously, and the most significant changes appeared when K shifted from 1 to 2 (Figure 2A). A sharp peak in ΔK was also observed at *K* = 2 (Figure 2B). Thus, the association panel was divided into two sub-groups, designated P1 and P2 (Figure 2C). Rapeseed accessions with Q matrix values < 0.6 or ≥0.6 were assigned to the P1 or P2 sub-groups, respectively, resulting in 105 lines for the former and 263 lines for the latter (Table S1, Data S2). The analysis of relative kinship showed that 56.8% of between-line kinship coefficients equaled 0, and 95.5% kinship coefficients ranged from 0 to 0.2 (Figure 2D, Data S2). Thus, most lines in the panel were very weakly related.

### Association mapping for salt tolerance indices and SNC

**Figure 3:**
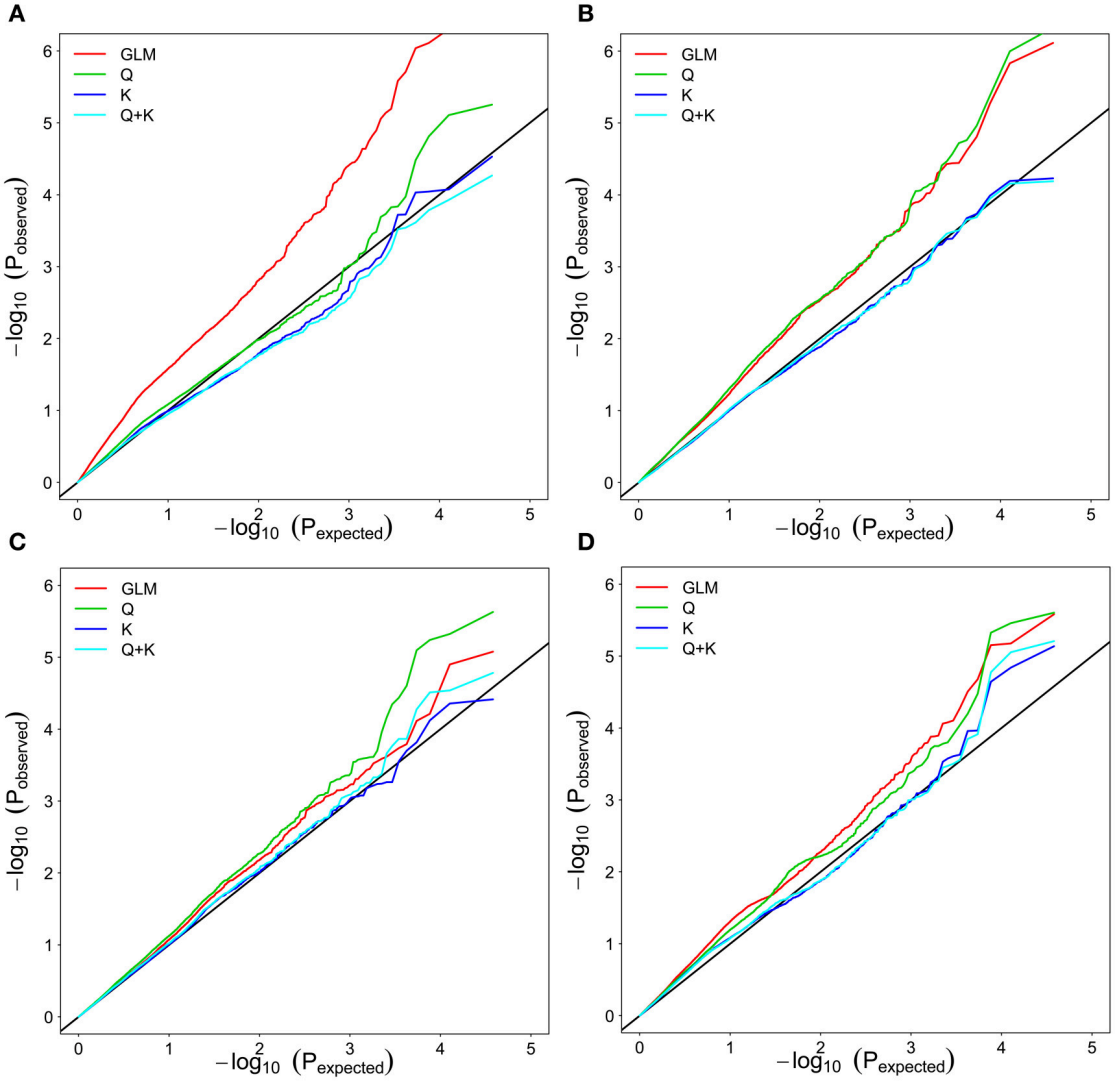
**Quantile–quantile plots of estimated−log_10_(P) from association analysis of associated traits in rapeseed cultivars. (A)** ST-RL. **(B)** ST-SL. **(C)** ST-SFW. **(D)** SNC. GLM considering no Q and K; Q: GLM considering Q; K: MLM considering K; Q + K: MLM considering Q and K; ST, salt tolerance index; RL, root length; SL, shoot length; SFW, shoot fresh weight.

To determine the most appropriate model (see Genome-Wide Association Analysis) for a GWAS, model fit was tested with salt tolerance traits (ST-RL, ST-SL, ST-SFW, and SNC) (Figure 3). Based on quantile–quantile (Q-Q) plots, the best-fit model varied depending on rapeseed traits. For example, the Q, K, and Q + K models were a better fit for the expected *P*-values of ST-RL (Figure 3A). The K and Q + K models were a better fit for the expected *P*-values of ST-SL (Figure 3B). All four models were good fits for expected *P*-values of ST-SFW (Figure 3C). The Q, K, and Q + K models were a better fit for the expected *P*-values of SNC (Figure 3D). To make the most efficient use of genotypic and phenotypic data in this study, the Q and Q + K models were applied for the association analysis.

**Figure 4:**
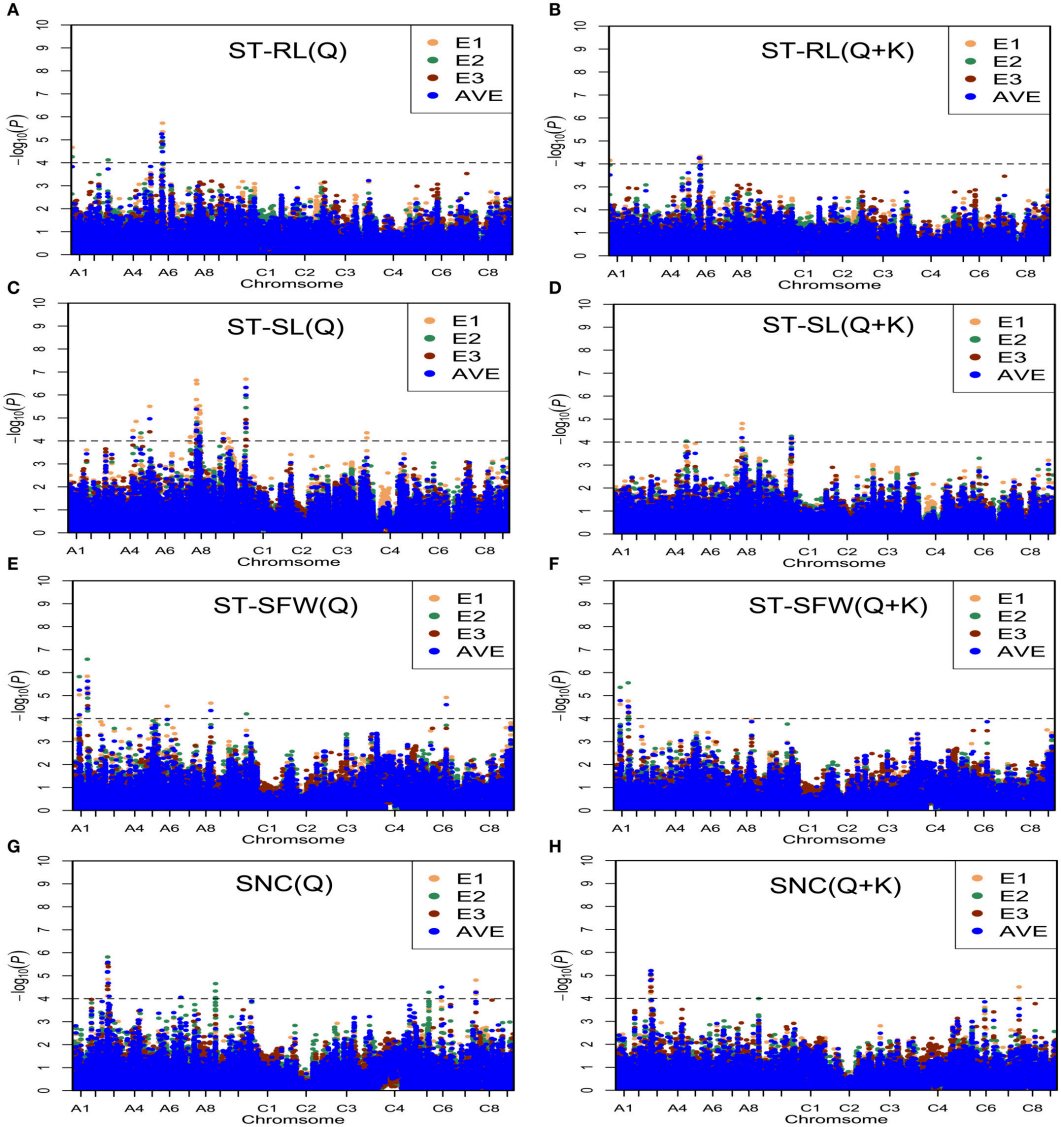
**Manhattan plots generated from genome-wide association analysis results for salt tolerance related-traits using Q and Q + K models. (A)** ST-RL (Q). **(B)** ST-RL (Q + K). **(C)** ST-SL (Q). **(D)** ST-SL (Q + K). **(E)** ST-SFW (Q). **(F)** ST-SFW (Q + K). **(G)** SNC (Q). **(H)** SNC (Q + K). E1, greenhouse in 2013; E2, greenhouse in 2014; E3, natural environment condition in 2014; AVE, average values across three environments. Gray horizontal line depicts significance threshold (*P* < 0.0001).

With these two models, we identified 75 significantly associated (*P* < 0.0001 or −Log(P) > 4.0) SNPs for the salt-related traits ST-RL, ST-SL, ST-SFW, and SNC (Figure 4, Table S4). Among the 75 associated SNPs, 58 SNPs were identified in greenhouse in 2013 (E1); 29 SNPs were identified in greenhouse in 2014 (E2); 10 SNPs were identified in natural environment in 2014 (E3); 34 SNPs were identified using average values (AVE) across three environments (Table S4). In addition, 32% (24/75) of the SNPs were identified in more than one growth environment, indicating their high reliability. Additionally, six, 47, 10, and 12 SNPs were distributed on chromosomes A1, A3, A4, A5, A6, A7, A8, A9, A10, C1, C4, C5, C6, and C7.

To integrate putative QTLs, we modified the method as previously described (Mohammadi et al., 2015; Liu et al., 2016). If the lead SNP and following SNPs were within 1 Mb, or the LD statistic pairwise *r*^2^ between lead SNP and the following SNPs was > 0.35, these SNPs were regarded as identifiers for the same QTL. Finally, these 75 significant SNPs constituted 25 QTLs and explained 4.21–9.23% of the phenotypic variation among the rapeseed accessions (Table 3, Table S5).

**Table 3:** **SNPs significantly associated with salt tolerance indices and SNC in the three growth environments**.

**Trait**	**SNPs^a^**	**Chr**	**Site (b)**	**Allele**	**MAF**	**Q**		**Q + K**		**Env^c^**
						**−Log_10_P**	**R^2^(%)^b^**	**−Log_10_P**	***R*^2^(%)^b^**	
ST-RL	Bn-A01-p1372430	A01	973485	A/G	0.135	4.67	5.96	4.15	5.67	E1
						4.26	5.37			E2
	Bn-A03-p9873327	A03	9088303	T/C	0.41	4.12	5.59			E1
						4.12	5.54			E2
	Bn-A06-p3066372	A06	2959211	A/G	0.16	5.73	7.44	4.33	6.69	E1
						4.95	6.27			E2
						5.11	6.5			AVE
ST-SL	Bn-A04-p11164883	A04	12315774	A/G	0.305	4.45	5.65			E1
						4.15	5.24			AVE
	Bn-A04-p14778146	A04	15229011	A/G	0.395	4.85	6.17			E1
	Bn-A05-p1744534	A05	1887184	A/C	0.465	4.16	4.27			E1
	Bn-A05-p2246038	A05	2366787	A/C	0.304	4.14	4.24			E1
						4.35	4.46	4.04	4.3	E2
	Bn-A05-p17420613	A05	14469584	T/C	0.419	5.5	7.69			E1
						4.39	6.02			E3
						4.96	6.88			AVE
	Bn-A07-p15589001	A07	17485098	T/C	0.15	4.18	4.78			E1
	Bn-A09-p33179406	A09	30892924	T/C	0.17	4.32	5.56			E1
	Bn-A08-p3269074	A08	2746242	T/C	0.117	6.48	8.04	4.81	6.24	E1
						4.81	5.8			E2
						4.72	5.71			AVE
	Bn-Scaffold000211-p65635	A10	4230491	T/G	0.467	4.11	4.21			E1
	Bn-scaff_15838_1-p1804241	C01	2159899	T/C	0.424	6.69	9.23	4.09	6.06	E1
						5.89	8.03	4.25	6.23	E2
						4.93	6.68			E3
						6.33	8.71	4.16	6.19	AVE
	Bn-scaff_15908_1-p535407	C04	5694092	A/G	0.259	4.35	5.19			E1
ST-SFW	Bn-A01-p7574355	A01	6916594	T/C	0.276	5.03	6.2	4.61	6.02	E1
						5.82	7.26	5.36	7.05	E2
						5.24	6.48	4.78	6.31	AVE
	Bn-A01-p21855770	A01	18441121	T/C	0.212	5.84	7.53	4.77	6.43	E1
						6.58	8.49	5.55	7.75	E2
						5.32	6.81	4.54	6.21	AVE
	Bn-A06-p7703242	A06	7333685	T/G	0.23	4.54	4.71			E1
	Bn-A08-p20091818	A08	17365577	T/C	0.173	4.67	5.51			E1
						4.35	5.09			AVE
	Bn-A10-p15741819	A10	15808392	A/G	0.477	4.2	5.59			E2
	Bn-scaff_16116_1-p406330	C06	28299662	T/G	0.191	4.92	5.15			E1
						4.6	4.78			AVE
SNC	Bn-A03-p8032849	A03	7321761	A/G	0.266	4.12	4.85			E1
						5.81	7.04	5.19	6.37	E2
						4.56	5.45	4.48	5.29	E3
						5.46	6.6	5.05	6.11	AVE
	Bn-A09-p13614414	A09	10135644	T/G	0.411	4.65	4.81			E2
	Bn-scaff_16268_1-p640088	C05	12621782	A/G	0.493	4.28	5.52			E2
	Bn-scaff_18206_1-p435713	C06	18701952	A/G	0.362	4.07	5.39			E2
						4.2	5.59			AVE
	Bn-scaff_16110_1-p3586046	C07	41328003	T/C	0.213	4.81	5.74	4.49	5.42	E1

a *Only the lead SNPs at a defined locus are shown*.

b *Percentage of phenotypic variance explained by the lead SNP*.

c *E1 greenhouse in 2013, E2 greenhouse in 2014, E3 natural environment in 2014, AVE, average values across three environments*.

**Figure 5:**
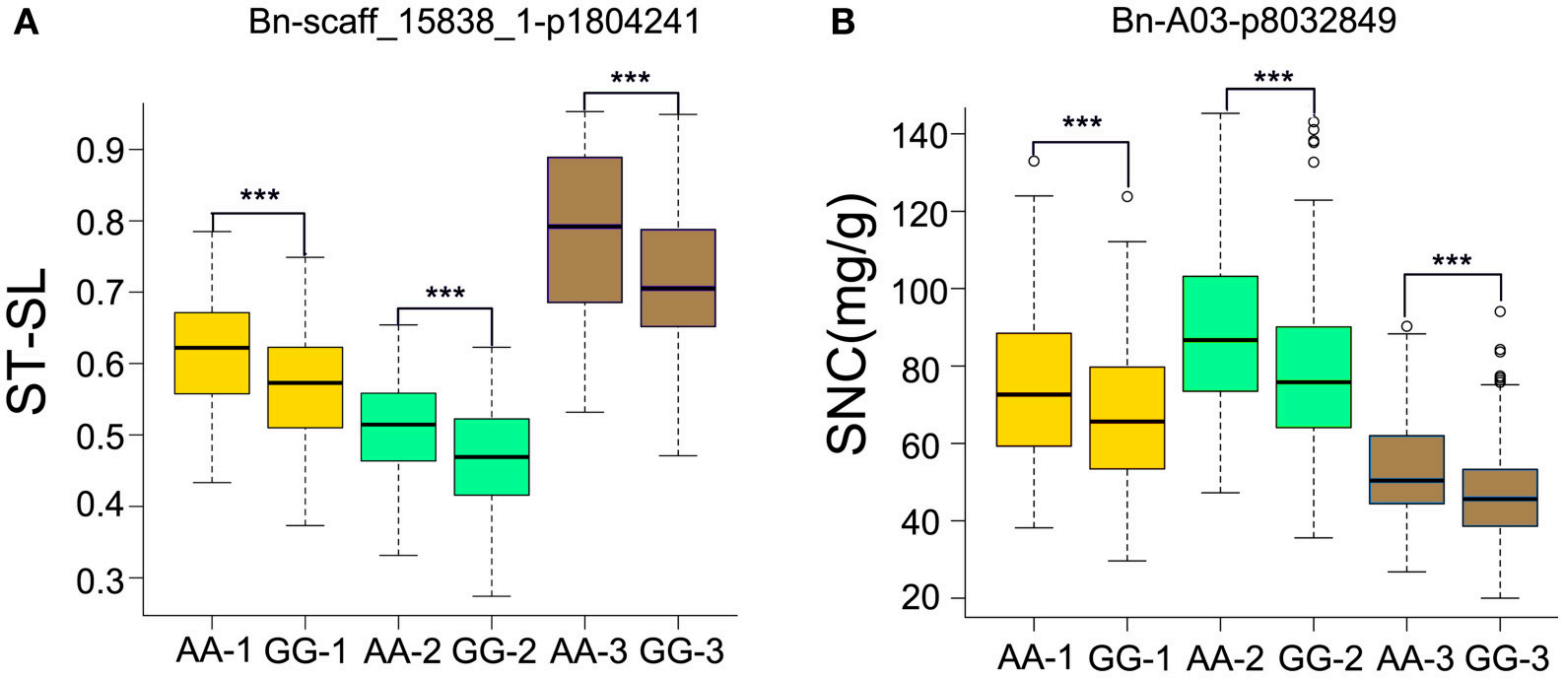
**Phenotypic differences between lines with different alleles of the SNPs associated with SNC and ST-SL in three growth environments. (A)** *Bn-A03-p8032849* associated with ST-SL. **(B)** *Bn-scaff_15838_1-p1804241* associated with SNC. The numbers in brackets behind AA or GG refer to the three growth environments. ^***^P < 0.001.

For ST-RL, six significantly associated SNPs, constituting three QTLs, were identified on chromosomes A1, A3, and A6 and explained 5.37–7.44% of the phenotypic variation (Table 3, Table S4). Among these six SNPs, five were validated in more than one growth environment. For ST-SL, 47 significantly associated SNPs (constituting 11 QTLs) were distributed on the A4, A5, A7, A9, A10, C1, and C4 chromosomes and explained 4.21–9.23% of phenotypic variation. Among them, 11 SNPs were validated in more than one individual environment (Table 3, Table S4). For ST-SFW, 10 significantly associated SNPs (constituting six QTLs) were identified on A1, A6, A8, A10, and C6 chromosomes and explained 4.39–8.49% of the phenotypic variation observed (Table 3, Table S4). Among them, five were validated in more than one individual environment. For SNC, 12 significantly associated SNPs (five QTLs) were identified on A3, A9, C5, C6, and C7 chromosomes and explained 4.69–7.04% of the phenotypic variation (Table 3, Table S4). Among these, three SNPs were validated in all tested environments. The SNP Bn-scaff_15838_1-p1804241 (ST-SL) and *Bn-A03-p8032849* (SNC) were identified both in three environments and using average values across three environments (Table 3). In order to know the impact of these stable QTLs on phenotype. We found that ST-SL of accessions carrying the AA allele of *Bn-scaff_15838_1-p1804241* was significantly higher (*P* < 0.001) than those with the GG allele in all three environments (Figure 5A). The SNC of accessions carrying the AA allele for *Bn-A03-p8032849* was significantly higher (*P* < 0.001) than those with the GG allele in all three environments (Figure 5B).

### Predicting salt-tolerant genes in rapeseed cultivars

**Figure 6:**
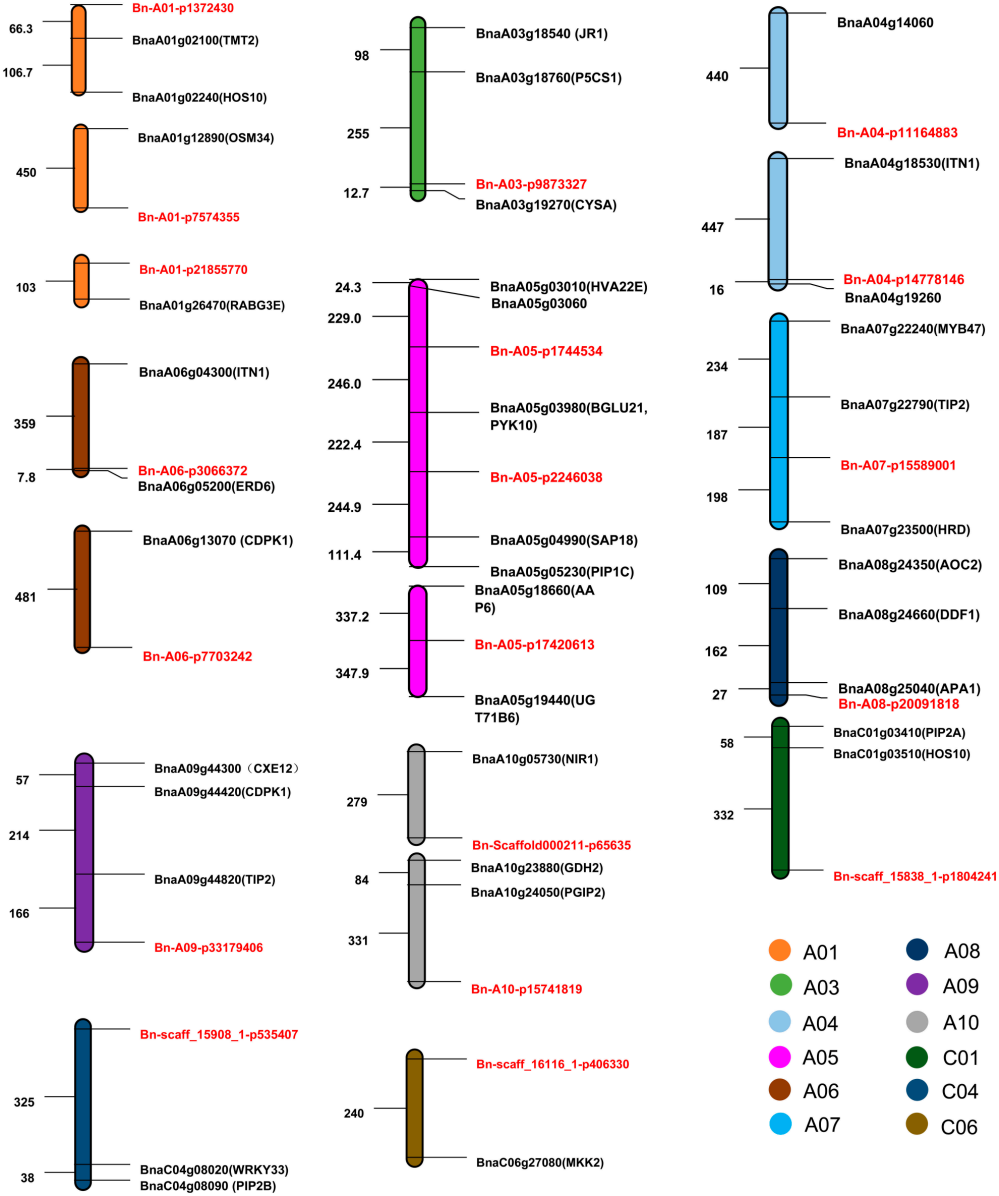
**The distribution pattern of candidate genes and their corresponding SNPs associated with salt tolerance (ST)**. The abbreviations of orthologous genes in *Arabidopsis thaliana* are shown in brackets after the candidate genes. SNPs are marked in red. Numbers represent the relative distances in the genome, 1 = 1 kb.

To predict candidate genes related to ST-RL, ST-SL, ST-SFW, and SNC, we identified *B. napus* genes which were the orthologous to *A. thaliana* salt stress-related genes located within the 500 kb of peak SNPs. Based on the *B. napus* reference genome (Chalhoub et al., 2014), we obtained 38 candidate salt stress-related genes (Figure 6, Table S6). The physical distance between these candidate genes and the significant SNPs varied from 8 to 490 kb (Figure 6). Among these genes, *BnaA01g02240, BnaA01g02100, BnaA03g18540, BnaA03g18760, BnaA03g19270, BnaA06g04300*, and *BnaA06g05200* were identified for ST-RL; they are genes which were the orthologous to the *Arabidopsis* genes *HOS10, TMT2, JR1, P5CS1, CYSA, ITN1*, and *ERD6*, and located within 500 kb of peak SNPs *Bn-A01-p1372430, Bn-A03-p9873327*, and *Bn-A06-p3066372*. For ST-SL, 22 genes (including *BnaA04g18530, BnaA05g03980, BnaA05g05230, BnaA05g04990, BnaA07g22240, BnaA07g22790, BnaC01g03510*, and *BnaC04g08090*) were identified within 500 kb of peak SNPs (e.g., *Bn-A04-p11164883, Bn-A04-p14778146, Bn-A05-p2246038, Bn-A07-p15589001, Bn-A09-p33179406, Bn-scaff_158381-p1804241*, and *Bn-scaff_159081-p535407*); they are genes which were the orthologous to *Arabidopsis ITN1, PYK10, PIP1C, SAP18, MYB47, TIP2, HOS10*, and *PIP2B*. For ST-SFW, nine genes (*BnaA01g12890, BnaA01g26470, BnaA06g13070, BnaA08g24350, BnaA08g24660, BnaA08g25040, BnaA10g23880, BnaA10g24050*, and *BnaC06g27080*) were identified within 500 kb of the peak SNPs (*Bn-A01-p7574355, Bn-A01-p21855770, Bn-A06-p7703242, Bn-A08-p20091818, Bn-A10-p15741819*, and *Bn-scaff_161161-p406330*); they are genes which were the orthologous to the *Arabidopsis* genes *OSM34, RABG3E, CDPK1, AOC2, DDF1, APA1, GDH2, PGIP2*, and *MAPKK2*. Unfortunately, we did not find candidate genes related to SNC.

## Discussion

### Population structure and model comparison

Association mapping has become a critical tool for identifying alleles and loci responsible for agronomically important traits. Its success, however, depends largely on species, target traits, marker density, and study population (Zhu et al., 2008). Spurious associations may result because genotypes of association populations from the same area may be more closely related than those from different areas (Zhao et al., 2007). Thus, the critical first step before initiating association mapping is to collect a wide range of germplasm resources with different genetic backgrounds. In the current study, we chose from a worldwide rapeseed collection to map loci responsible for salt-stress response and related growth, with the ultimate goal of discovering useful candidate genes for improving salt tolerance in plants. Our collection consisted of 368 rapeseed genotypes originating from 10 countries on four continents, indicative of a sample population with sufficient genetic variety for a feasible GWAS. Population structure (Q) and kinship (K) are major factors leading to false positive results in association analyses. To reduce the risk of false positives, we used a corrected MLM that considered both Q and K matrices, which was more effective than the Q or K alone (Stich et al., 2008). However, in some cases, the MLM (Q + K) model was too strict, and important SNPs could not be detected. Thus, other better models should be developed and used in future research.

### QTL for salt tolerance indices and shoot Na^+^ content

The salt tolerance index is considered a reliable measure for evaluating salt tolerance that controls for background effects among different genotypes (Long et al., 2013; Kan et al., 2015; Yong et al., 2015). We evaluated the salt tolerance indices of rapeseed seedlings for RL, SL, and SFW. Our results suggested that root and shoot salt-tolerance mechanisms may be different; ST-SL and ST-SFW were significantly and positively correlated with each other (*P* < 0.01), but ST-RL was not significantly correlated with either ST-SL or ST-SFW (Table 2).

Unraveling the genetic factors controlling salt tolerance in plants is a challenge due to the complexity of adaptive mechanisms that involve various pathways and molecular components (Long et al., 2013). To date, although some studies exist that focus on salt tolerance in *B. napus* (Farhoudi and Saeedipour, 2011; Jian et al., 2016; Zhao et al., 2016), but the studie on salt tolerance-related QTL are limited. To our knowledge, only one study like ours had been performed previously: association mapping of 85 inbred rapeseed lines identified 62 QTL for salt tolerance, shoot biomass, and ion homeostasis-related traits (Yong et al., 2015). In the present study, we identified 20 QTL associated with salt tolerance indices and five QTL associated with SNC in different regions of the *B. napus* genome (Table 3). Among these 25 QTL, 40% (10/25) were detected in more than one environment, and 60% (15/25) were detected in a single environment. These data indicate that significant interactions exist between QTL and growth environments. No co-associated QTL, however, was observed between any two salt tolerance indices, suggesting that the salt tolerance genetic mechanisms are complex and tissue-specific (Wang et al., 2011). Furthermore, QTLs distribution revealed that salt tolerance in *B. napus* is exclusively attributable to the A (19 QTLs) and C (6 QTLs) chromosomes.

The most important function of plant roots is to absorb water and nutrients from the soil. Thus, roots are the primary site for sensing abiotic stress from soil, including salinity and drought. In many circumstances, root sensitivity to stress limits the productivity of the entire plant (Ruan et al., 2010). Thus, understanding salt tolerance mechanisms in roots is essential to improve plant salt resistance. In our study, the three QTLs, *Bn-A01-p1372430, Bn-A03-p9873327*, and *Bn-A06-p3066372* root-related were identified on A1, A3, and A6 chromosomes, respectively, and were detected in more than one environment. Furthermore, 11 QTL associated with ST-SL were identified and four QTLs *Bn-scaff_15838_1-p1804241, Bn-A05-p2246038, Bn-A05-p17420613*, and *Bn-A08-p3269074* were validated in more than one individual environment. Remarkably, *Bn-scaff_15838_1-p1804241* was detected in all three environments, indicating that it is a stable QTL not easily affected by environmental change. No QTL associated with ST-RL and ST-SL was reported previously, thus all above QTLs identified in our study should be the novel QTLs. Additionally, six QTLs associated with ST-SFW were identified. In contrast to a previous study (Yong et al., 2015), we found that *Bn-A06-p7703242* was 2.39 Mb away from the salt tolerance-related 3075557d, suggesting that association signals detected in our study are valid. And Bn-A01-p7574355, Bn-A01-p21855770, Bn-A08-p20091818, Bn-A10-p15741819, Bn-scaff_16116_1-p406330 should be the novel QTLs associated with ST-SFW.

Although shoot Na^+^ content was not correlated with salt tolerance in rapeseed seedlings, the mechanism for Na^+^ uptake and exclusion still appeared to have some possible effects on rapeseed salt tolerance. We identified five QTLs associated with SNC on A3, A9, C5, C6, and C7 chromosomes. Notably, *Bn-A03-p8032849* was detected in all three environments and was only 244 kb away from the SNC-related SNP 3099118d identified previously (Yong et al., 2015), suggesting that *Bn-A03-p8032849* could be a reliable site affecting shoot Na^+^ content. Most identified QTLs have not been reported in previous studies and can be considered novel. They thus provide new references for the study of salt tolerance mechanisms and the breeding of salt-tolerant *B. napus* cultivars. Compared with the research published by Yong et al. (2015), the number of rapeseed accessions in our study was larger (368 to 85) and the NaCl concentration was higher (230–100 mM). Therefore, our results can provide a reference for the genetic mechanism of rapeseed salt tolerance under a relatively high salt concentration. Besides, another important point for the current study is that the experiments were conducted both at the natural condition and greenhouse but the experiment of Yong et al. (2015) was only conducted in greenhouse.

### Salt tolerance in *B. napus* L. is not linked to Na^+^ content in shoots

In many crop species such as durum wheat (Munns et al., 2012), barley (Nguyen et al., 2013), and rice (Lin et al., 2004), salt tolerance is significantly and negatively correlated with Na^+^ content in shoots. These plants have developed diverse strategies to resist salt stress, such as restricting Na^+^ uptake in root, activating shoot Na^+^ exclusion or cellular compartmentalization of excessive in root and shoot (Zhu, 2000; Shi et al., 2002), and K^+^ retention in root (Chen et al., 2005) and mesophyll (Wu et al., 2013). Nevertheless, our results indicate that the salt tolerance indices in rapeseed were not linked to shoot Na^+^ content, suggesting that the salt tolerance mechanism in rapeseed is more complicated and possibly influenced by multiple factors. Our results are consistent with previous work showing no significant correlation between salt tolerance index and leaf Na^+^ content (Yong et al., 2015). Plants evolved a variety of mechanisms in response to salt stress, including tissue tolerance to toxic ions, tolerance to osmotic stress, ROS scavenging, and beneficial ion homeostasis (e.g., K^+^, Ca^2+^) (Leshem et al., 2006; Shabala et al., 2006; Munns and Tester, 2008), and these different mechanisms probably combine to affect plant salt tolerance. Future comprehensive studies of rapeseed salt tolerance should introduce additional indicators of salt stress responses, such as malonaldehyde (MAD), chlorophyll content, and photosynthetic rate (Moradi and Ismail, 2007; Ellouzi et al., 2011). Nonetheless, our results provide an important first step to the study of salt tolerance mechanisms in rapeseed.

### Salt stress-related candidate genes

Currently, little is known about the genes involved in *B. napus* salt tolerance. However, many reports have dissected the molecular mechanisms of salt tolerance in *Arabidopsis* (Qiu et al., 2002), rice (Lin et al., 2004), and other crop species (Misra and Dwivedi, 2004). The candidate genes identified in our study can be split into several functional groups, including transcription factors, aquaporin, transporters, and enzymes. Most of these candidate genes have known orthologs in *Arabidopsis* and multiple crops.

Transcription factors include *HOS10*, encoding a nuclear localized R2R3-type MYB transcription factor; *MYB47*, a member of the R2R3 factor gene family; *HRD* and *DDF1*, both part of the DREB subfamily within A-4 of ERF/AP2 transcription factors; and *WRKY33*, a WRKY transcription factor. R2R3-type MYB transcription factor is involved in stress responses to cold, osmotic imbalance, and salt. The *hos10-1* mutant plants are extremely sensitive to NaCl and freezing temperatures (Zhu et al., 2005). Similarly, the link we found between *MYB* genes and salt tolerance in rapeseed is corroborated in previous research. *OsMYB2*-overexpressing rice is more tolerant to salt, cold, and dehydration stresses, but more sensitive to abscisic acid than wild-type rice. In addition, overexpressing wheat *TaMYB30-B* in *Arabidopsis* improves drought stress tolerance during the germination and seedling stages (Zhang et al., 2012). *AtWRKY33* (*WRKY DNA-binding protein 33*) is a transcription factors that participate in various stress responses, including against salt and drought (Mare et al., 2004; Pandey and Somssich, 2009). Loss-of-function analysis of *wrky33* mutants showed a moderate increase in NaCl sensitivity, and *WRKY33* overexpression increased *Arabidopsis* NaCl tolerance (Jiang and Deyholos, 2009).

*PIP1C, PIP2A, PIP2B*, and *TIP2* are aquaporins (AQPs) that act as water channels and respond to water deprivation and salt stress, which has two subfamilies—the plasma membrane intrinsic proteins (PIP) and tonoplast intrinsic proteins (TIP) (Alexandersson et al., 2005). *PIP1C* is a member of PIP subfamily PIP1, while *PIP2A* and *PIP2B* are members of PIP2. *TIP2* is a member of the tonoplast intrinsic protein (TIP) family. Overexpressing rice *PIP* genes in wild-type *Arabidopsis* enhanced tolerance to salt and drought (Guo et al., 2006).

Next, transporter proteins include *TMT2*, a tonoplast monosaccharide transporter 2; *ERD6*, a putative sucrose transporter; and AAP6, a high affinity amino acid transporter. The expression of *ERD6*, which encoded a putative sugar transporter of *Arabidopsis*, was up-regulated by dehydration and cold treatment (Kiyosue et al., 1998).

Additionally, *P5CS1, CXE12, CDPK1, RABG3E, AOC2, GDH2, MAPKK2*, and *PGIP2* belong to the enzyme functional group. Previous studies have shown that proline accumulation frequently occurs in plants under salt stress and can improve the salt tolerance of plants (Nanjo et al., 1999; Jimenez-Bremont et al., 2006). *P5CS1* encodes a delta-1-pyrroline-5-carboxylate synthase that catalysis the rate-limiting step in proline biosynthesis. *P5CS1*-knockout lines exhibit lowered proline synthesis, resulting in salt hypersensitivity and *AtP5CS1* expression was induced under salt stress in *B. napus* (Xue et al., 2009). *CXE12* and *CDPK1* encode a protein carboxylesterase and a calcium-dependent protein kinase, respectively. Plant CDPKs may function as calcium sensors and are important to the regulation of plant growth and development, as well as to biotic and abiotic stress responses (Zou et al., 2010). *RABG3E* encodes a small GTPase involved in membrane trafficking. Hydrogen peroxide induces *AtRADG3E* expression, and transgenic plants expressing this gene exhibited increased tolerance to salt and osmotic stresses, as well as reduced ROS accumulation during salt stress (Mazel et al., 2004). *AOC2* encodes allene-oxide cyclase. *GDH2* encodes the beta-subunit of the glutamate dehydrogenase. *MKK2* (*MAP kinase kinase 2*) is a regulator of MPK6 and MPK4 in response to cold and salt stresses. *Arabidopsis* plants overexpressing *MKK2* exhibited constitutive *MPK4* and *MPK6* activity, as well as heightened expression of stress-induced marker genes, all of which increased freezing and salt tolerance. In contrast, *mkk2* null plants are hypersensitive to salt and cold stress (Teige et al., 2004). Cold and salinity can induce *MKK2* expression in oilseed rape, suggesting that *MKK2* is important for the regulation of plant response to those stresses. In addition, *OsMKK6* overexpressing lines exhibit higher salt stress tolerance in rice (Kumar and Sinha, 2013). *PGIP2* (*polygalacturonase inhibiting protein 2*), a gene that encodes a polygalacturonase inhibiting protein involved in plant defense response. Abiotic stress (e.g., cold, NaCl, dehydration) upregulates *BrPGIP2* in Chinese cabbage leaves (Hwang et al., 2010).

In addition to the above mentioned genes, some other genes were identified related to salt tolerance. *At HVA22E*, which is up-regulated to varying degrees in response to cold, salt, ABA treatment, or dehydration (Chen et al., 2002). *HARDY* is a part of the stress-related *AP2/ERF* gene family. Overexpression of *AtHARDY* improved drought and salt tolerance in rice (Karaba et al., 2007) and *Trifolium alexandrinum* L (Abogadallah et al., 2011).

Interestingly, most of the salt tolerance-related genes we identified were related to osmotic tolerance, whereas candidate genes related to ion stress were not found. Previous studies have reported that plants can experience NaCl stress gradually or in one step, leading to either salt stress or salt shock, respectively (Shavrukov, 2013). Salt shock is an extreme salt stress, wherein plants are exposed suddenly to high salinity levels. Gene expression patterns differ in response to salt stress and salt shock. Salt stress initiates relatively fewer expression changes in osmotic stress tolerance-related genes and more in ionic stress tolerance-related genes, whereas salt shock leads to the opposite outcome. Thus, our study provides a valuable reference for the study of both salt tolerance and osmotic tolerance mechanisms in *B. napus*. Future studies should examine these candidate genes to determine their functional characteristics in relation to rapeseed salt tolerance.

## Author contributions

JS, TF, and JW conceived and designed the study. BY, CM, and JT provided advice on the experimental design. HW, LC, JG, and QL performed phenotype measurements. HW and LC analyzed all data. HW wrote the manuscript. All authors reviewed and edited the manuscript.

## Funding

This research was financially supported by the National Key Research and Development Program of China (grant number 2016YFD0101300) and the Program for Modern Agricultural Industrial Technology System (grant number CARS-13).

### Conflict of interest statement

The authors declare that the research was conducted in the absence of any commercial or financial relationships that could be construed as a potential conflict of interest. The reviewer MZ and handling Editor declared their shared affiliation, and the handling Editor states that the process nevertheless met the standards of a fair and objective review.
