# Supplementary figures and images for: Genome-Wide Association Study Reveals the Genetic Architecture Underlying Salt Tolerance-Related Traits in Rapeseed (Brassica napus L.)
Source: Front Plant Sci. 2017 Apr 26;8:593. doi: 10.3389/fpls.2017.00593 (PMC5405135; doi:10.3389/fpls.2017.00593)

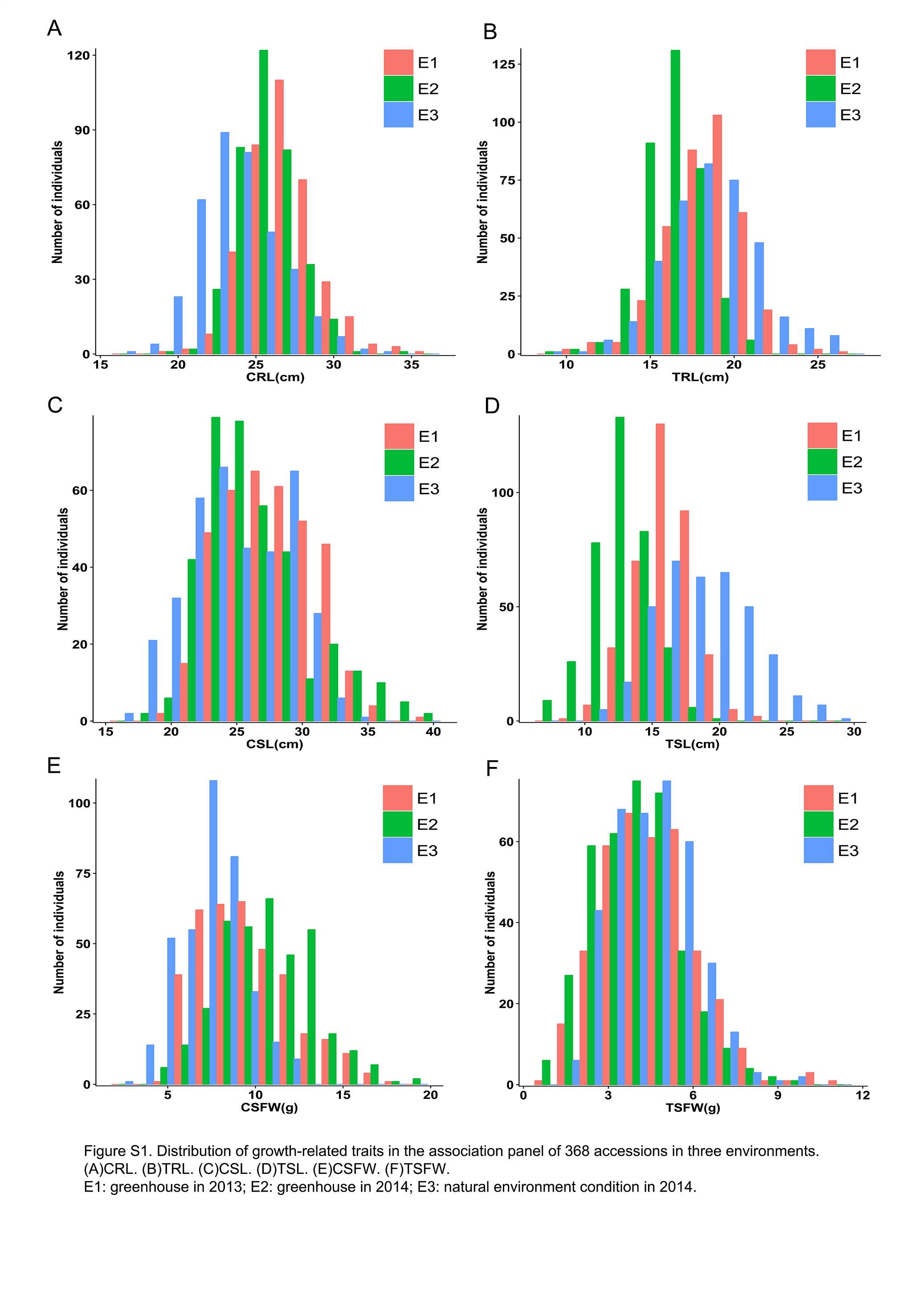

Supplement: Supplementary file 9 [file Image1.TIF]
